# Established and Emerging Concepts to Treat Imbalances of Iron Homeostasis in Inflammatory Diseases

**DOI:** 10.3390/ph11040135

**Published:** 2018-12-11

**Authors:** Verena Petzer, Igor Theurl, Günter Weiss

**Affiliations:** 1 Department of Internal Medicine II, Medical University of Innsbruck, 6020 Innsbruck, Austria; verena.petzer@i-med.ac.at (V.P.); igor.theurl@i-med.ac.at (I.T.); 2 Christian Doppler Laboratory for Iron Metabolism and Anemia Research, Medical University of Innsbruck, 6020 Innsbruck, Austria

**Keywords:** Anemia of chronic disease, anemia of inflammation, hepcidin, anti-hepcidin therapy, iron supplementation

## Abstract

Inflammation, being a hallmark of many chronic diseases, including cancer, inflammatory bowel disease, rheumatoid arthritis, and chronic kidney disease, negatively affects iron homeostasis, leading to iron retention in macrophages of the mononuclear phagocyte system. Functional iron deficiency is the consequence, leading to anemia of inflammation (AI). Iron deficiency, regardless of anemia, has a detrimental impact on quality of life so that treatment is warranted. Therapeutic strategies include (1) resolution of the underlying disease, (2) iron supplementation, and (3) iron redistribution strategies. Deeper insights into the pathophysiology of AI has led to the development of new therapeutics targeting inflammatory cytokines and the introduction of new iron formulations. Moreover, the discovery that the hormone, hepcidin, plays a key regulatory role in AI has stimulated the development of several therapeutic approaches targeting the function of this peptide. Hence, inflammation-driven hepcidin elevation causes iron retention in cells and tissues. Besides pathophysiological concepts and diagnostic approaches for AI, this review discusses current guidelines for iron replacement therapies with special emphasis on benefits, limitations, and unresolved questions concerning oral versus parenteral iron supplementation in chronic inflammatory diseases. Furthermore, the review explores how therapies aiming at curing the disease underlying AI can also affect anemia and discusses emerging hepcidin antagonizing drugs, which are currently under preclinical or clinical investigation.

## 1. Introduction

Iron has a crucial role in all living organisms. In humans, iron is essential for many biochemical processes, including electron transfer reactions in mitochondria, the citric acid cycle, gene expression, binding and transport of oxygen, regulation of cell growth and differentiation as well as the cellular immune response [1]. From a systemic point of view, hepcidin, a liver-derived hormone, has been found to be the master regulator of iron homeostasis, controlling cellular iron efflux [2]. Hepcidin binds to the sole known iron exporter, ferroportin (FPN), mediating internalization and degradation of this transporter [3,4]. As a further consequence, dietary iron absorption as well as iron release from cells, such as macrophages, is prevented [5]. Hepcidin expression is regulated by different stimuli, such as anemia, hypoxia, and inflammation [6]. Different molecular pathways involved in hepcidin expression have been uncovered [7,8]. Among these, the bone morphogenetic protein (BMP)-SMAD signaling pathway is the most critical. Liver endothelial cell-derived BMP6 and BMP2 have non-redundant roles to induce hepcidin expression. However, BMP6 is the dominant ligand and a threshold signaling of BMP6 via the BMP-SMAD pathway is indispensable for sufficient and appropriate hepcidin induction [9,10,11,12]. During inflammation, hepcidin expression is induced via the interleukin (IL)6-JAK-STAT and Activin B-SMAD1/5/8 signaling pathways [2,13,14,15]. As FPN regulates iron release from absorptive enterocytes in the duodenum and from iron recycling macrophages of the mononuclear phagocyte system (MPS), elevated hepcidin levels during inflammation cause diminished systemic iron availability [16]. While iron retention in the MPS appears to be beneficial for host responses during infections, as it withholds this metal from invading extracellular pathogens, anemia is an undesired, ultimate consequence of iron restriction in patients suffering from chronic diseases [17,18,19]. Consequently, anemia of inflammation (AI) or anemia of chronic disease (ACD) represents the most common disease-related complication in patients suffering from rheumatoid arthritis (RA) inflammatory bowel diseases (IBD), cancer, infectious diseases, and chronic kidney disease (CKD) [1,20,21,22,23,24,25,26].

Whereas the development and persistence of anemia in several diseases, including infections and cancer, has been associated with a poor prognosis, the true impact of alterations in iron homeostasis or anemia on the pathology of the underlying disease remains largely elusive [27]. However, anemia negatively impacts on many aspects of the patients [28]. Moreover, iron exerts multiple effects on immune cell differentiation, functionality, and plasticity, which has been studied in depth toward the interconnection of iron homeostasis with the biology of M1 and M2 macrophages [29,30]. M1 macrophages, being either activated upon pathogen recognition or stimulated by cytokines (e.g., interferon-gamma (IFNγ), tumor necrosis factor-alpha (TNFα), or IL1, IL6 and IL10), induce subtle changes of transcellular iron fluxes, aiming to limit the availability of the essential nutrient iron for circulating pathogens. Therefore, the term, “nutritional immunity”, has been proposed [31]. Thereafter, cytokines directly or cytokine-inducible products, such as oxygen radicals and nitric oxide, regulate the expression of critical iron transport and storage proteins [19,32,33,34,35,36,37,38]. Consequently, the accessibility of iron for microbes is modulated and their growth and pathogenicity is impacted. Moreover, bacteria can acquire iron from holo-transferrin. Therefore, limitation of circulating transferrin-bound iron levels and mutations of the iron binding sites of transferrin were shown to be protective against infections with circulating bacteria [39,40,41].

Of note, iron per se also affects immune effector pathways of macrophages and, subsequently, T-cell differentiation by regulating IFNγ activity, nitric oxide formation, or T-helper cell plasticity [42,43,44,45,46]. Thus, local and systemic iron availability determines not only microbial growth, but also the efficacy of anti-microbial immune effector pathways. It appears that the alterations of systemic and macrophage-responsible iron fluxes are specifically regulated depending on the nature and localization of the pathogen [19,32,33,39,47]. The hepcidin–FPN axis has attracted specific attention regarding alterations of iron fluxes during infections. Hepatocytes produce large amounts of hepcidin following challenge with circulating bacteria, resulting in iron retention with the MPS and low circulating iron levels [17,18]. Moreover, autocrine formation of hepcidin by macrophages further reduces cellular iron availability for circulating pathogens [19,39,48,49]. In contrast, invasion of cells and macrophages with bacteria, such as *Listeria*, *Mycobacteria,* or *Salmonella*, induces alternative mechanisms. Specifically, the upregulation of FPN by different mechanisms results in macrophage iron efflux and limitation of bacterial growth [50,51,52,53,54]. In addition, M1 macrophages and other immune cells produce several factors, such as lipocalin-2, lactoferrin, and calprotectin, which limit the bacterial access to iron [55,56,57]. Of note, in addition to iron flux regulation by the FPN-hepcidin axis, several hepcidin-independent mechanisms have been identified that control iron trafficking during infection [33,50,58,59,60,61].

In contrast, M2 macrophages exert anti-inflammatory effects and these cells are highly specialized for iron recycling from senescent erythrocytes via erythrophagocytosis, yielding approximately 90% of the daily needs of iron for erythropoiesis [30]. Iron is released from heme by the anti-inflammatory enzyme, heme oxygenase-1 [62,63]. The latter enzyme has attracted specific interest because it exerts immune regulatory effects, but, importantly, it also exerts disease tolerance during certain infections by limiting tissue damage, thereby improving the outcome from sepsis [64,65].

While it has long been known that iron is essential for the production of hemoglobin of red blood cells, our knowledge on the regulation of iron homeostasis under steady state conditions and in association with different pathologies has dramatically expanded over the past centuries thanks to the identification and characterization of numerous iron genes and associated regulatory molecules [1]. Indeed, unbiased iron supplementation or withdrawal therapy via phlebotomy dates to the middle ages. However, due to our expanding knowledge on iron metabolism regulation during inflammation, targeted modulation of specific iron metabolic pathways, including the hepcidin-FPN axis, has emerged only recently [3,66,67]. Although we have several established and novel iron therapies at hand, there are still many unresolved questions and unmet needs when treating imbalances of iron homeostasis in patients with inflammatory diseases. This includes lack of gold-standard tests to properly distinguish between absolute versus functional ID, lack of knowledge regarding safe and efficient therapeutic start and end points as well as complications of iron redistribution and supplementation strategies towards the course of the diseases underlying AI.

## 2. Diagnosis

The diagnosis of AI is based on several laboratory markers. Classically, hemoglobin levels are decreased; markers of inflammation, such as C-reactive protein (CRP) or IL6, are increased; and iron homeostasis is altered as follows: Circulating iron levels are low, transferrin saturation (Tf-Sat) is reduced, and ferritin concentrations are normal or increased (Table 1) [68]. Diagnosis becomes challenging if AI is associated with true ID (AI/ID), as there is still a lack of a gold standard for differentiation between AI and AI/ID. However, as therapies to overcome anemia differ, proper diagnosis and understanding of underlying pathophysiological regulations are necessary [69]. While ferritin strongly correlates with the body’s iron stores in IDA, ferritin levels are not reliable during inflammation. Thus, low ferritin levels (<30 mg/mL) in any case indicate true ID, but ferritin levels are upregulated during inflammation largely independently of iron availability [70]. This fact has led to corrections towards elevated cut-off values for ferritin during concomitant inflammation [71,72]. Until now, the gold standard for diagnosis of ID is still the microscopic evaluation of iron-stained bone marrow aspirates, which is not routinely used due to its high invasiveness [73]. A recent study in heart failure patients proposed to use serum iron and Tf-Sat instead of ferritin to diagnose true ID, which was evaluated by bone marrow staining [74]. Compared to ferritin-based definition of ID (with a sensitivity and specificity of 82% and 72%, respectively), the diagnosis of ID based on reduced Tf-Sat (cut-off: ≤19.8%) and low serum iron (cut-off: ≤13 μmol/L) had an improved sensitivity (94%) and specificity (84% and 88%) in this specific group of patients. Although these findings need further confirmation among other disease entities, it highlights that ferritin-based definitions of ID appear to be suboptimal.

As erythrocytes are the main consumers of iron and thus most affected by ID, efforts have been undertaken to establish markers that are related to red blood cell morphology and iron content of these cells. Alongside the well-established classical hematological indices of the mean corpuscular volume (MCV) and mean corpuscular hemoglobin (MCH), new parameters, such as the hemoglobin content of reticulocytes, percentage of hypochromic red blood cells, and the soluble TfR (sTfR), were introduced as indicators of iron availability for the erythron and/or efficacy of erythropoiesis [68,75,76]. Some studies recommend the sTfR as an alternative biomarker to distinguish between absolute (or true) and functional ID. In general, absolute ID and higher rates of erythroid output causes an up-regulation of the TfR on erythrocytes, which then concomitantly leads to higher detectable forms of its cleaved monomer, the sTfR, in the plasma [77]. As inflammation negatively impacts erythropoiesis and TfR expression, sTfR values are also altered during inflammation [26,36]. Therefore, the use of this marker led to unsatisfactory sensitivity and specificity (83% and 50%, respectively) for the detection of ID compared to bone marrow findings in a cohort of 180 anemic children in Mozambique [78]. Attempts to correct this marker for inflammation, using a sTfR versus log ferritin ratio, did not classify patients properly, thus limiting the diagnostic potential of this test [79]. While these parameters add additional information on true iron availability for erythropoiesis in patients with AI, none of these measurements are adjudged as efficient for distinguishing between AI and AI/ID.

A number of reports indicating that hepcidin is competent to distinguish between IDA and AI in several diseases, including RA, anemia of cancer, anemia of critical illness, and IBD, have suggested hepcidin to be a promising biomarker in the future [16,80,81,82,83]. Moreover, other reports also exist suggesting that plasma hepcidin levels could predict the response to oral iron in different settings [84,85,86,87]. However, there are also reports from studies in hemodialysis patients to the contrary, highlighting the need for further detailed investigations [88,89,90]. Further discussion on hepcidin is presented in Section 3.2.2. The measurements of molecules that affect hepcidin expression under different conditions may turn out to be of diagnostic benefit. Erythroferron, hypoxia inducible factors (HIFs), and platelet derived growth factor BB are all signaling peptides induced by hypoxia and were found to impact directly or via modulation of hepcidin on iron availability for erythropoiesis [91,92,93]. The biomarkers of hypoxia thus hold promise to better identify subjects suffering from AI/ID and to predict the erythroid response in patients with AI with and without ID, once commercially available ELISAs are available [94,95,96]. Of importance, none of these tests is currently standardized, which is a necessity to make them a reliable routine biomarker for the evaluation of iron status. Consequently, trials investigating these parameters cannot be easily compared, making interpretations even more difficult. However, according to a recent report, a hepcidin reference standard allows equivalence and comparability between hepcidin measurement results [97].

Despite ongoing efforts to find and establish new biomarkers, a recently published study conducted in a cohort of IBD patients revealed that differentiation between AI and iron deficiency anemia (IDA) and the combination thereof was only possible in 22% of all anemic patients, because only CRP, hemoglobin, and ferritin levels were available as diagnostic markers. [98]. This highlights that improvement of diagnostic approaches to identify patients with true ID in the setting of inflammation is urgently needed and is still a challenging field of investigation.

## 3. Treatment Strategies

Treatment of ID and IDA is paramount as it is associated with several detrimental effects on quality of life, exercise capacity, mental status, and activity of patients [99,100]. To this end, two strategies can be pursued. First, treatment of the underlying disease; second, if a cure cannot be achieved, therapies directly or indirectly addressing imbalances of iron homeostasis are indicated.

### 3.1. “First line”: Treatment of the Underlying Inflammation

If possible, treatment of the underlying disease is decidedly the pivotal approach to treat AI. Resolution of inflammation results in the normalization of hepcidin levels, leading to the correction of macrophage iron retention and normalization of duodenal iron uptake. In addition, the negative cytokine-mediated proliferative effects on hematopoiesis are abrogated, overall leading to anemia improvement. One approach, which has been shown to be effective, is the neutralization of inflammatory cytokines. Accordingly, targeted therapy using an anti-IL6 receptor antibody (Tocilizumab) improved anemia in patients suffering from multicentric Castleman’s disease (MCD), a lymphoproliferative disorder where IL6 was found to be the main cytokine contributing to its pathogenesis [101,102]. Of note, IL6 is one major driver for hypoferremia in patients suffering from AI [2]. Further work-up revealed that anemia amelioration due to IL6 receptor blockade is related to down-regulation of hepcidin levels [103,104]. In parallel, a monoclonal anti-IL6 antibody (Siltuximab) has also been evaluated for its potential to decrease hepcidin plasma levels and consequently improved anemia not only in patients suffering from MCD, but also in subjects with multiple myeloma and solid tumors [105,106,107].

Of interest, not only systemic, but also autocrine hepcidin expression in macrophages has been found to be of importance in AI and possibly also for iron distribution in cancer cells [48,108]. In patients with ovarian cancer, polarization towards an M1 phenotype and high IL6 levels were associated with more profound anemia. Treatment with Tocilizumab resulted in the reversion of iron restriction and improvement of anemia, supporting previous evidence that anti IL6-directed therapy may be effective for anemia in cancer [108,109].

TNFα is also a target to treat the underlying complications and ameliorate anemia. Monoclonal antibodies directed against TNFα (e.g., Infliximab, Adalimumab, Golimumab) are routinely applied in patients suffering from RA and IBD. As TNFα’s contribution to AI is different from IL6, the beneficial effect on anemia was ascribed to discontinuation of TNFα’s negative impact on bone marrow erythropoiesis or, likewise, erythrocyte’s half-life, without having direct effects on hepcidin levels [15,110,111,112,113,114]. However, a study investigating two different TNFα inhibitors in IBD patients found that the beneficial effect of anti-TNFα is indirect and it is mediated via down-regulation of IL6 [115]. Anti-TNF therapy may also reduce intravascular radical formation, thereby preventing the radical-mediated damage of erythrocyte membranes and increasing their circulating half-life. Moreover, comparative evaluation of TNFα inhibitors and Tocilizumab revealed that IL6-mediated therapy, directly affecting hepcidin levels, is more effective than TNFα inhibitors in respect to anemia correction [116]. Furthermore, hematological response after one year of anti-TNFα treatment was only observed in 34% of patients, even with oral iron supplementation [117].

Patients suffering from myeloproliferative neoplasms (MPN) have been shown to develop anemia, in part as a consequence of elevated hepcidin levels [118]. As mutations related to the activity of Janus kinase 2 (Jak2), resulting in constant activation, were found to be central to the pathogenesis of MPN, Jak2 inhibitors became one treatment option However, erythropoietin (EPO) is an essential hormone for sufficient production of red blood cells and also signals via the JAK2 pathway [119]. Consequently, anemia dose-dependently developed in patients who were treated with a JAK2 inhibitor (Ruxolitinib) and this was a dose-limiting adverse event [120,121]. In contrast, results from a phase II study for the treatment of myelofibrosis with a different Jak2 inhibitor (Momelotinib) surprisingly resulted even in an improvement of anemia [122]. Further dissection of the underlying mechanisms demonstrated that Momelotinib not only effectively inhibited Jak2 signaling, but also blocked ACVR1/ALK2-driven induction of hepcidin, resulting in an egress of iron from macrophages to sites of erythropoiesis [123].

Although these therapies are effective in lowering hepcidin levels and therefore ameliorate the anemia seen in chronic diseases, these therapies are probably not be suitable for sole treatment of AI because of potential side effects of these therapies, such as increased risk of infections due to impaired host responses [124]. A compromise might be a combinatorial therapeutic approach to target both the improvement of iron status and the treatment of infections.

### 3.2. Iron Supplementation and Iron Redistribution Therapies

Despite ongoing development of new treatment strategies and efforts towards personalized-based medicine, diseases, such as cancer, chronic heart failure, autoimmune diseases, and end stage kidney diseases, are proving unattainable because of persistent chronic inflammation. This being the case, anemia must be addressed via different approaches. Besides direct iron supplementation, iron redistribution strategies are emerging. The choice of the most appropriate therapy depends on the categorization of anemia whether there is pure AI with functional ID versus AI in combination with true ID. While iron replacement therapy appears to be mandatory in the latter setting, iron supplementation is questionable in patients with pure AI and strategies aiming at iron redistribution from macrophages to the circulation may be the more pragmatic approach.

#### 3.2.1. Iron Supplementation

In general, iron can be directly supplemented either via the oral or intravenous (i.v.) route. However, this decision is based on several factors, including the availability and cost of drugs, the underlying disease, the degree of inflammation, therapeutic efficacy, and side effects, but also on patients’ compliance and convenience (Table 2). Oral iron may be used in ID and mild to moderate anemia, specifically among patients with a stable disease or only a low grade of inflammation [14,15,69,125]. Oral iron may also be effective in patients with AI and combined true ID due to the fact that ID-mediated inhibition of hepcidin expression dominates over inflammation-driven hepcidin induction [126,127]. Indications when i.v. iron therapy should be initiated are not that straight forward, based on the low grade of available evidence, and heterogeneity between guidelines for different disease entities [128]. However, i.v. iron may be used if oral iron therapy is ineffective, causes therapy-related side effects, and in patients with impaired oral iron absorption (Table 2). Of note, guidelines for recommendations whether to use oral or iv iron supplementation vary in different countries, in particular with regard to CKD. Examples include the Canadian guidelines, the Caring for Australians with Renal Impairment (CARI), the National Institute for Health and Care Excellence (NICE), and the Kidney Disease: Improving Global Outcomes (KDIGO), with each of them having their own guidelines and diagnostic algorithms, as well as choice of preferred administration route (oral vs. i.v.) [129,130,131,132]. This situation is far from being satisfactory as it causes deterrence and confusion among physicians and highlights the necessity of prospective clinical outcome data from rigorously conducted randomized controlled trials.

Nevertheless, the importance of i.v. iron supplementation among CKD patients became clear when the first human EPO preparation was licensed for use in dialysis-associated anemia nearly 30 years ago. Patients who suffered from EPO hypo-responsiveness experienced resolution of this condition with concomitant administration of i.v. iron. Hence, KDIGO guidelines propose that iron therapy should be aimed to treat ID, increase iron stores prior to initiation of therapy with erythropoiesis stimulating asgents (ESA), and enhance the response to these drugs [129].

In addition, within the last few years, concerns regarding the use of ESA (including EPO) for the treatment of anemia in CKD patients have been raised [133,134]. This was because of increased risk of adverse clinical outcomes, such as stroke and venous thromboembolic disease, culminating in high mortality [113,114,115,116]. Indeed, the US Food and Drug Administration (FDA) released a black box warning on the use of high EPO doses. Consequently, iron supplementation, either alone or in combination with ESA agents, are recommended as front-line options [129,130,131,132]. The latter is also related to findings of the TREAT (Trial to Reduce Cardiovascular Events with Aranesp^®^ Therapy) study, which not only emphasized the possible risks related to ESA therapy, but also revealed that iron therapy increases hemoglobin levels and is capable of delaying the initiation need of ESA therapy [133,134]. Furthermore, another multicenter, prospective, and randomized study, FIND-CKD (Ferinject^®^ assessment in patients with IDA and non-dialysis-dependent chronic kidney disease), reported that both i.v. and oral iron supplementation were capable of maintaining hemoglobin levels, thus reducing the dosages of ESA [135]. However, a recent randomized trial in non-dialyzed patients with CKD found that the use of i.v. iron was associated with an increased risk for adverse cardiovascular events and infections when compared to oral iron treatment [136].

Iron supplementation in patients with IBD is also still far from being consistent and many questions are still open, including the value of iron supplementation in subjects without anemia, or the preferred route of iron supplementation. Anemia seen in IBD is unique, as most patients suffer from AI together with ID, which is the consequence of continuous blood loss by the inflammatory mucosa and impaired iron intake as a consequence of malnutrition [125,137,138]. I.v. iron, as a sole treatment, has been shown to correct anemia in more than 80% of patients [139]. According to the European Crohn’s and Colitis Organisation (ECCO) guidelines published in 2015, iron supplementation is recommended whenever IDA is present. In contrast to recommendations made for CKD patients, iron supplementation aims to normalize hemoglobin levels in patients with IBD [140]. I.v. iron is recommended in patients with a clinically active disease, previous intolerance to oral iron, severe anemia (Hb < 10 g/dL), and who have initiated combination therapy with an ESA. Otherwise, oral iron therapy may be applied. However, several comparative studies, where i.v. versus oral iron supplementation was investigated, revealed that in IBD patients with AI and true ID and low disease activity, oral iron is as effective as i.v. iron to correct anemia [141,142,143,144,145,146]. Although AI is listed as a common cause for “non-IDA” in IBD subjects, no guidelines are provided regarding the practice of iron supplementation for these patients. Indeed, data from clinical trials on this issue are scarce. However, in view of published evidence that IBD patients with anemia have higher CRP values and a more active disease status, the necessity for further evaluation of this eventual relationship and its clinical management is evident [147,148].

In conclusion, no matter which subtype of AI is present, today’s evidence and treatment recommendations are based on altered biomarkers of haematology and inflammation and their correction, while end-point data on the effects of iron therapies (e.g., death, survival and disease resolution or progression) are almost completely lacking. Moreover, hardly any information from prospective trials is available regarding optimal therapeutic targets (e.g., hemoglobin or ferritin levels), which, however, may be different according to the underlying disease. One pioneering study (PIVOTAL for Proactive IV Iron Therapy in Hemodialysis Patients) addressed some of these end-points (risk of death, major adverse cardiovascular events, and infection) in patients undergoing hemodialysis and has just been published [149,150]. A high-dose regimen of i.v. iron (400 mg of iron sucrose per month, administered in a proactive fashion) was compared to a low-dose regimen (0 mg–400 mg of iron sucrose per month, administered in a reactive fashion). While the high iron regimen led to a reduced cumulative dose of administered ESA, there was no association with any of the end-points. How this study will influence existing guidelines and iron supplementation strategies remains to be seen.

#### 3.2.2. Hepcidin Modulation

As mentioned above, hepcidin is the master regulator of systemic iron homeostasis, as this hormone is decisive for FPN expression, regulating iron efflux [151]. Thus, circulating levels of hepcidin determine the transfer of iron from the diet via the duodenum and release of iron from macrophages of the MPS. Since hepcidin is central to the pathophysiology of AI, several strategies that either modulate the synthesis of hepcidin or neutralize its activity have been developed [152,153]. The purpose of hepcidin modulation is to reverse iron retention in the MPS, thus enhancing iron availability for erythropoiesis in AI. As multiple causes lead to AI (e.g., negative impact of cytokines on erythropoiesis, impaired EPO activity), it needs to be investigated if increase of iron availability on its own is sufficient to effectively restore hemoglobin levels. Alternatively, a combination with an ESA could lead to a more favorable outcome [25,154,155,156,157,158].

The first approach that has been used were antibodies directed against hepcidin, which initially were only effective to reverse anemia in animal models when combined with ESA, whereas a subsequently developed human antibody modulated iron homeostasis in mice and cynomolgus monkeys without concomitant ESA administration [159].

Another approach for hepcidin neutralization is based on the use of antichalins (bioengineered lipocalin; small ligand-binding protein) or aptamers (also called Spiegelmer or Lexaptepid pegol L-stereoisomeric RNA aptamer). Indeed, these compounds have also been proven to be effective in preclinical models, and phase I trials have been successfully completed. In detail, positive data were obtained from a phase I study for the anticalin PRS-080 thus a phase II study was initiated, which is evaluating the effect of PRS-080 administration in anemic hemodialysis CKD patients (https://clinicaltrials.gov/ct2/show/NCT03325621) [160]. The outcomes of this clinical study on anti-hepcidin treatment are awaited. In addition, details on the impact of this compound on iron metabolism in cynomolgus monkeys has just recently been published [161]. In parallel, a placebo-controlled study on the safety, pharmacokinetics, and pharmacodynamics of the spiegelmer NOX-H94 in healthy humans demonstrated that hepcidin was inhibited dose-dependently, thus causing an increase in serum iron and Tf-Sat [162]. Furthermore, clinical phase II studies for the treatment of AI in patients suffering from multiple myeloma, low grade non-, or Hodgkin lymphoma, and ESA-hypo-responsive chronic hemodialysis patients have shown favorable effects, but cohorts were small, so further assessment is warranted [163,164].

As BMPs, specifically BMP2 and BMP6, are potent inducers of hepcidin, inhibition of the BMP-SMAD pathway is an attractive therapeutic approach to control hepcidin production [10,11,12,165]. Since this pathway is highly complex, involving different players, many possible targets can be contemplated [166]: First, BMP sequestration is one strategy. Therefore, BMP6 antibodies, a soluble hemojuvelin–Fc fusion protein, and modified heparins have been developed [167,168,169,170]. A phase I clinical trial of such a latter compound (Roneparstat, SST0001), which has competitive heparanase inhibitor properties, has been conducted in patients suffering from multiple myeloma, regarding its anti-myeloma effect, dosing, and safety profile (https://clinicaltrials.gov/ct2/show/record/NCT01764880) [171]. Impacts on hepcidin and iron metabolism have not been published yet.

Representing one step further down the BMP/SMAD pathway, efforts have been undertaken to target the BMP receptor (BMPR). TP-0184, a small-molecule inhibitor of ALK2 activity, has entered a phase I study this year (https://clinicaltrials.gov/ct2/show/NCT03429218), after having shown promising effects on hepcidin suppression in vitro and in preclinical mouse models [172]. Not only BMPR, but also BMP co-receptors, have been investigated as hepcidin lowering strategies. Two monoclonal antibodies targeting hemojuvelin have been developed, and are still in preclinical development [173].

A third reasonable approach to counteract hepcidin activity is to block hepcidin-induced internalization of FPN. Even though a phase II trial for such a stabilizing FPN antibody has been successfully completed in 2015, its further development has been stopped [174,175].

Notably, EPO at high doses can decrease hepcidin levels [176]. This effect is only of short duration and seems to be indirect, as signals derived from expanding erythroid progenitors in the bone marrow mediate this suppression [177,178]. Indeed, among CKD patients, no long-term effects of EPO on hepcidin levels have been observed, which, however, may also be partly related to impaired urinary hepcidin excretion [179]. In addition, HIF-prolyl hydroxylase inhibitors (HIF-PHIs), stabilizing HIFs, and thus activating HIF-controlled pathways, such as intrinsic EPO expression, have been reported to impact on iron homeostasis [93,180]. However, these effects can be traced back to transcriptional regulation, resulting in enhanced expression of specific iron transporters in the intestine (such as FPN and divalent metal transporter 1), thereby promoting iron absorption. Table 3 gives an overview of the drugs that directly or indirectly modulate hepcidin levels.

## 4. Perspectives

Anemia, being the final consequence of imbalances in iron homeostasis, in the setting of chronic diseases must be recognized as a clinical condition contributing to the morbidity of patients and awareness for ID must be improved. Indeed, due to the knowledge gap in clearly defining and diagnosing this condition, IDA, AI, and combined AI/ID are often used mutually. However, ID itself precedes anemia and should be detected, even outside the context of anemia. Efforts to counteract this common misconception have been made and a group of experts has proposed the following overarching definition for ID: “Iron deficiency is a health-related condition in which iron availability is insufficient to meet the body’s needs and which can be present with or without anemia” [181]. Of importance, ID is not only associated with anemia, but also higher morbidity and mortality among certain chronic diseases, which could be alleviated after treatment [100,182,183,184,185]. For example, in patients suffering from chronic heart failure, ID has been shown to adversely impact performance status and quality of life, including prolonged hospitalizations independent of anemia [186,187,188]. Another significant upcoming challenge will be the management of anemia of the elderly, which often has a heterogeneous and multifactorial etiology, but is also specifically related to age-related changes [189].

Although AI is a condition that should be treated, there is also an evolutionary rational for iron restriction during inflammation: Iron restriction is beneficial during acute infections, especially to withhold iron from circulating microbes [19,39]. Malaria represents one of the best studied examples in this context [65,190,191]. The fact that red blood cells are the host for plasmodia highlights their dependency on iron metabolism. An important finding was just recently made to better understand underlying pathomechanisms: FPN expression on red blood cells is critical to prevent detrimental intracellular iron accumulation and hemolysis, all in all leading to a more severe course of malaria. Of interest, these authors found that a human mutation in *FPN* (Q248H), which is unresponsive to hepcidin-mediated degradation, has been positively selected in sub-Saharan African populations [192]. Thus, anti-hepcidin treatment strategies as listed above could be discussed as a treatment option for malaria in the future. Despite anemia being associated with this infection, iron supplementation has been shown to be detrimental. This is also in line with studies showing that iron supplementation in children of developing countries resulted in higher morbidity and mortality from infections [193,194].

Moreover, there is increasing evidence for the role of iron availability for the gut microbiome and oral versus i.v. iron have different effects on the composition of the microbiome [143,195,196]. This is of interest, because the composition of the gut microbiome was found to play decisive roles for the progression of IBD and carcinogenesis in different mouse models [197]. Further workup in vitro showed that certain iron formulations (ferric citrate and ferric ethylenediaminetetraacetic acid) also bear the risk of exacerbation of colon cancer advancement in an amphiregulin-dependent fashion, however, it needs to be defined whether or not the dosages used in such models are relevant for humans [198].

Another issue of general importance are the effects of iron supplementation or hepcidin targeting strategies on immune regulation. This is based on the observation that iron impacts on the differentiation and proliferation of immune cells, but also directly impacts on immune effector pathways either by promoting oxygen radical formation or inhibiting pro-inflammatory cytokine production or anti-microbial immune effector pathways of macrophages [29,199,200]. Pre-clinical and clinical models have shown that iron supplementation reduces TNFα formation in CKD patients while negatively impacting on the host response in mammalian models of invasive fungal infection [44,201]. Thus, depending on the underlying disease, iron supplementation could have disease modifying effects through its regulatory effects on the immune function [44,202].

## 5. Conclusions

Anemia and ID in the setting of chronic inflammatory diseases are leading causes of morbidity worldwide. While we have gained significant knowledge on the mechanism underlying iron misdistribution and development of AI, highlighting the role of immune mediators and the iron hormone hepcidin, there is still the need for reliable biomarkers to evaluate iron homeostasis in patients suffering from inflammatory diseases and to choose the best therapy or to predict its efficacy. Specifically, distinction between AI versus AI combined with true iron deficiency is of importance because these groups of patients may likewise need different iron redistribution therapies. The development of new drugs (e.g., hepcidin antagonists) and the improvement of old drugs (novel formulation for oral and intravenous iron preparations) are the subject of future investigations. Although there is good evidence that iron supplementation improves quality of life, the effect of iron supplementation on the course of an underlying disease or associated co-morbidities are poorly understood. There is only limited information on therapeutic start- and end-points for iron supplementation and anemia correction in such patients. However, negligence of anemia and iron deficiency may also exacerbate the underlying disease state and cause clinical deterioration [203,204]. Thus, there is still a lot to learn to optimize and personalize treatment in subjects with AI. Therefore, investigations through pre-clinical models, but also through prospective randomized trials, are urgently needed to gain more detailed insights into this clinically very frequent, but poorly understood condition.

## Figures and Tables

**Table 1:** Diagnostic markers for the diagnosis of different types of inflammatory anemia.

Marker	Anemia of Inflammation	Anemia of Inflammation plus Iron Deficiency Anemia	Limitations/Comments
Bone marrow iron staining	Normal–Elevated	Normal–Reduced	Gold standard Invasive method, not routinely used
Serum Iron	Low	Low	Underlies diurnal variations
Ferritin	Elevated	Reduced–Normal–Elevated	Most commonly used marker Ferritin is an acute phase protein and does not accurately reflect iron status during inflammation Ferritin < 30 ng/mL always associated with true iron deficiency
Transferrin	Normal–Reduced	Normal–High	
Tf-Sat	Low	Low	Dependent on iron and transferrin levels
sTfR	Normal–Elevated	Elevated	Good marker for needs of iron for erythropoiesis in absence of inflammation Values affected by inflammation and ESA application
sTfR/log Ferritin	Normal	Elevated	Used for differentiation, but there is a lack of a prospective study
Hepcidin	Elevated	Normal–Reduced	Expression is more affected by iron deficiency (suppressing) than by inflammation Not standardized Weak correlations in CKD patients Possible predictive parameter for success of iron and/or ESA treatment
Erythroferron	Not known	Not known	Not standardized Higher ERFE levels in CKD patients Positively correlated with serum erythropoietin and negatively with hemoglobin
MCV/MCH	Normal	Normal–Reduced	If reduced, indication of iron deficiency
Reticulocyte Hb content	Normal–Reduced	Reduced	Indicated insufficient iron availability for erythropoiesis, not prospectively studied
Hypochromic RBC	Normal	Normal–Elevated	Related to MCV, as a sensitive marker for iron availability for erythroid progenitors Cut-off values are different between different machines
CRP	Increased	Increased	Non-specific inflammatory marker Iron–independent parameter Correlation with severity of anemia
IL6	Increased	Increased	Non-specific inflammatory marker Iron–independent parameter

**Table 2:** Characteristics of oral and intravenous iron therapy.

	Indication(s)	Benefits	Limitations	Uncertainties/Comments
Oral iron	True iron deficiency Combined true and functional iron deficiency with low grade inflammation	Low costs Easy to apply Effective if applied appropriately	High pill burden Low bioavailability High rate of non-responders Ineffective in the presence of high hepcidin levels Gastro-intestinal side effects Low compliance	Identification of the underlying cause Absorption defect must be excluded No predictor for response Oral iron as a trigger for cancer or intestinal inflammation Effects on gut microbiome Disease specific therapeutic start and endpoints
Intravenous iron	True and functional iron deficiency Absorption defects Severe anemia Intolerance to oral iron therapy Lack of efficacy of oral iron therapy	Faster replacement of iron stores than with oral iron Fewer gastro-intestinal side effects New i.v. iron formulations allowing high single dose administration Effective in the presence of inflammation Better control of compliance	Rare but possible life threatening anaphylactic reactions Route of application requires consultation of a physician Higher costs Hypophosphatemia	Long-term outcome on underlying disease unclear No predictor of response Possible iron-induced oxidative/nitrosative stress Unknown efficacy in patients with more advanced inflammation and/or high hepcidin levels Disease specific therapeutic start and endpoints

**Table 3:** Drugs impacting on hepcidin-mediated alteration of iron homeostasis.

Name(s)	Primary Indication(s)	Target	Drug Type	Mechanism
Tocilizumab	Rheumatoid arthritis Systemic juvenile idiopathic arthritis Giant cell arteritis MCD Cytokine release syndrome	IL6R	Humanized monoclonal antibody	IL6 signaling inhibition
Siltuximab	MCD	IL6	Chimeric monoclonal Antibody	IL6 binding
Infliximab	IBD (Crohn’s disease, Ulcerative colitis) Rheumatoid arthritis Psoriatic arthritis Ankylosing spondylitis Psoriasis	TNFα	Chimeric monoclonal antibody	TNFα binding/blocker
Adalimumab	IBD (Crohn’s disease, Ulcerative colitis) Rheumatoid arthritis Psoriatic arthritis Ankylosing spondylitis Psoriasis Hidradenitis suppurativa Juvenile idiopathic arthritis	TNFα	Humanized monoclonal antibody	TNFα binding/blocker
Momelotinib GS-0387 CYT-387	Myelofibrosis	JAK1 and JAK2	Small molecule	Jak1 and Jak2 inhibition Blockig of hepcidin production via ALK2 inhibition
CSJ137	Hepcidin modulation Anemia amelioration	BMP6	Antibody	BMP6 binding/blocking
SST0001 RO-82 RO-68 NAc-91 NAcRO-00	Myeloma therapy Hepcidin modulation	BMP6	Modified heparin	BMP6 binding
TP-0184	Antitumor activity in advanced solid tumors Hepcidin modulation Anemia amelioration	ALK2	Small molecule	ALK2 inhibition
h5F9.23, h5F9-AM8	Hepcidin modulation Anemia amelioration	HJV/RGMc	Antibody	BMP Co-receptor binding binding
Spiegelmer Aptamers NOX-H94H	Hepcidin modulation Anemia amelioration	Hepcidin	Lexaptepid pegol L-stereoisomeric RNA aptamer	Hepcidin binding
PRS-080	Hepcidin modulation Anemia amelioration	Hepcidin	Antichalin, bioengineered lipocalin	Hepcidin binding
Erythropoetin	Anemia	EpoR	Protein	Induction of Erythroferron and blockage of hepcidin

## Abbreviations

ACD	Anemia of chronic disease
ACVR	Activin A receptor
AI	Anemia of inflammation
ALK	Activin receptor-like kinase
BMP	Bone morphogenic protein
BMPR	Bone morphogenic protein receptor
CKD	Chronic kidney disease
CRP	C-reactive protein
EPO	Erythropoietin
EPOR	Erythropoietin receptor
ERFE	Erythroferrone
ESA	Erythropoiesis stimulating agent
FDA	Food and drug administration
FPN	Ferroportin-1 AKA SLC40A1
Hb	Hemoglobin
HIFs	Hypoxia inducible factors
HIF-PHD	Hypoxia inducible factor prolyl hydroxylase inhibitors
IBD	Inflammatory bowel disease
ID	Iron deficiency
IDA	Iron deficiency anemia
IFNγ	Interferon gamma
IL	Interleukin
IL6R	Interleukin 6 receptor
i.v.	Intravenous
JAK	Janus kinase
MCD	Multicentric Castleman’s disease
MCH	Mean corpuscular hemoglobin
MCV	Mean corpuscular volume
MPN	Myeloproliferative neoplasms
MPS	Mononuclear Phagocyte system
RA	Rheumatoid arthritis
RBC	Red blood cell
SMAD	Homologues of Sma and Mad (mothers against decapentaplegic) proteins
STAT	Signal transducer and activator of transcription
sTfR	Soluble transferrin receptor
TfR	Transferrin receptor
Tf-Sat	Transferrin saturation
TNFα	Tumor necrosis factor alpha
